# Aberrant Growth in 5‐Year‐Old Children After Antibiotics in the First Week of Life

**DOI:** 10.1111/apa.70322

**Published:** 2025-10-07

**Authors:** Lisanne M. van Leeuwen, Gina J. van Beveren, Marieke A. G. Peeters, Dennis Souverein, Sjoerd Euser, Debby Bogaert, Marlies A. van Houten

**Affiliations:** ^1^ Department of Paediatrics Spaarne Hospital Haarlem the Netherlands; ^2^ Department of Paediatric Immunology and Infectious Diseases Wilhelmina Children's Hospital/University Medical Center Utrecht Utrecht the Netherlands; ^3^ Regional Public Health Laboratory Kennemerland Haarlem the Netherlands; ^4^ Centre for Inflammation Research, Institute for Regeneration and Repair University of Edinburgh Edinburgh UK

**Keywords:** anti‐bacterial agents, growth and development, neonatal early‐onset sepsis

## Abstract

**Aim:**

We examined the relationship between early‐life antibiotics, different regimens, and growth until age five years.

**Methods:**

Data from two parallel birth cohorts were analysed: 128 healthy term‐born children and 147 term‐born children who received antibiotics for suspected neonatal sepsis, randomised across three regimens: Amoxicillin+Cefotaxime, Augmentin+Gentamicin, Penicillin+Gentamicin. Until age five years, growth, environmental exposures, diet, and physical activity data were collected. Primary outcomes were weight‐for‐age, height‐for‐age, and weight‐for‐height *z*‐scores with early‐life antibiotic exposure and the regimen as determinants of interest.

**Results:**

The median antibiotic exposure duration was 3 days (interquartile range 2.4–5.5 days). Children exposed to early‐life antibiotics had on average 0.26 lower weight‐for‐height *z*‐scores over the first five years compared to unexposed controls (*p* = 0.014). Especially children treated with Augmentin+Gentamicin showed lower weight‐for‐height *z*‐scores, compared to unexposed controls (coefficient = 0.36; *p* = 0.013). Additionally, at age five years, higher birth weight percentiles were associated with higher weight‐for‐age, height‐for‐age and weight‐for‐height and weekly lemonade consumption was associated with higher weight‐for‐age *z*‐scores.

**Conclusion:**

Antibiotics in the first week of life are associated with lower weight‐for‐height up to age five years, with effects varying by treatment type. To explain these effects, further examination of antimicrobial‐induced early‐life microbiome perturbations and subsequent growth is needed.

**Trial Registration:**

International Clinical Trial Registry Platform (https://trialsearch.who.int/): NL4882 and NL3821

**Summary**

- While antibiotic use has been linked to altered growth, the long‐term effects of early‐life antibiotics and specifically those of different regimens remain unclear.
- Week 1 antibiotics are associated with reduced weight‐for‐height over the first 5 years, especially after early‐life amoxicillin/clavulanic acid and gentamicin exposure.
- Clinicians should consider the type of early‐life antibiotic regimen for early‐onset sepsis carefully, and diagnostic tools for appropriate initiation of antibiotics are required to prevent overtreatment.

**Abbreviations**
CI: Confidence Interval
GP: General Practitioner
MUIS: Mirobiome Utrecht Infant Study
ZEBRA: Zuigelingen En Bacteriele Resistentie na Antibiotica (*neonates and bacterial resistance after antibiotics*)

## 1 Introduction

Newborns are highly susceptible to invasive bacterial infections and a suspected early‐onset neonatal sepsis is the main reason for antibiotic prescription within the first week of life [1]. Diagnosing early‐onset sepsis is often challenging due to subtle symptoms at birth and the lack of reliable diagnostic tools. Therefore, although blood culture‐confirmed sepsis occurs in only 0.5–0.8 per 1000 newborns annually [2], up to 8% of term newborns in the European Union receive systemic antibiotics for a suspected early‐onset neonatal sepsis [3, 4], corresponding to 4000 treated newborns per year in The Netherlands alone [5].

Early‐life antibiotic use can affect various physiological systems that are still developing over the course of infancy. For example, the first days of life are important for establishing the infant's microbiome [6, 7, 8]. The infant's microbiome performs several functions that contribute to metabolic processes, the education and maturation of the immune system [9, 10] and providing colonisation resistance against pathogenic microbes. Specifically, the intestinal microbiome is essential in breaking down and utilising dietary components that contribute to host‐growth [11, 12, 13, 14]. Our group has previously shown that early‐life antibiotics disrupt the assembly of the neonatal gut microbiome composition up to at least one year of life [15], with decreased *Bifidobacterium* spp. and increased *Klebsiella* and *Enterococcus* spp. abundances in antibiotic‐treated infants compared to unexposed controls, which others confirmed up to age two years [16]. Additionally, the effects of antibiotics on gut microbiome composition differed between regimens, with Amoxicillin + Cefotaxime showing the largest effects on both microbial community composition and antimicrobial resistance gene profile, whereas Penicillin + Gentamicin exhibited the least [15].

Consequently, antibiotic‐induced microbial disruptions could result in long‐term alterations in growth patterns. Research on early‐life antibiotic exposure has primarily focused on (repeated) exposures beyond the neonatal period, and these studies reported an increased tendency towards overweight and obesity in antibiotic‐treated children, particularly when treated within the first 6 months of life [17, 18, 19, 20, 21]. Studies examining the effect of antibiotics in the neonatal period found that treatment was associated with decreased height and weight during the first year of life [16, 22] and in boys up to six years. Murine data further supports this, showing a weight reduction in male but not female mice after faecal microbiota transplants of antibiotic‐exposed neonates [16]. Nevertheless, the number of studies investigating the consequences of early‐life antibiotics on later life growth is still limited, with a lack of data on the differential effects between antibiotic regimens.

The primary aim of this study is to examine growth in children exposed to antibiotics in the first week of life. Second, to assess the impact of different antibiotic regimens on growth, and lastly to evaluate further factors contributing to growth characteristics at age five years.

## 2 Methods

### 2.1 Study Population

Data was collected as part of two birth cohorts; the Microbiome Utrecht Infant Study (MUIS) [23] and Zuigelingen En Bacteriële Resistentie na Antibiotica (neonates and bacterial resistance after antibiotics; ZEBRA) [15]. The MUIS‐cohort consists of 128 healthy term‐born children (≥ 37 weeks of gestation), included between 2012 and 2014 in The Netherlands and followed over the first five years of life. This study served as the control population for the ZEBRA‐cohort, which is a cohort of 147 term‐born children (≥ 36 weeks of gestation) from three hospitals in The Netherlands recruited between 2015 and 2016, who received broad‐spectrum antibiotics for a suspected early‐onset neonatal sepsis in the first week of life. Treatment for early‐onset sepsis was initiated based on Dutch national guidelines in place at the time of the study [5]. Children were randomised 1:1:1 to receive one of three commonly used broad‐spectrum antibiotic regimens: Penicillin + Gentamicin, Amoxicillin + Cefotaxime, or Augmentin (amoxicillin/clavulanic acid) + Gentamicin. Children with major congenital anomalies and severe perinatal complications were excluded from both cohorts.

### 2.2 Data Collection

Children in both cohorts were followed over the first five years of life, with four overlapping visits in the first year (at 1, 4, 6, and 12 months of age) followed by yearly visits until age five years. After the first year of life, caregivers were asked permission for continued follow‐up, where parents could opt for detailed annual follow‐up, follow‐up at age five years only, or no further participation.

At inclusion, parents filled in questionnaires regarding maternal health status and environmental exposures. During follow‐up, parents filled in questionnaires regarding growth (weight and height), medication use, and environmental exposures (including breastfeeding and in‐house smoke exposure). At age five years, parents filled in additional questionnaires about the child's physical activities and dietary habits, and data from general practitioners (GP) regarding visits and medication use was retrieved.

### 2.3 Outcomes and Determinants

Primary outcomes included weight‐for‐age, height‐for‐age, and weight‐for‐height *z*‐scores. *Z*‐scores were generated using the World Health Organization growth references of anthropometric data [24]. The main determinant of interest was early‐life antibiotic exposure, defined as systemic antibiotic exposure in the first week of life. The second parameter of interest was the antibiotic regimen (i.e., none, Penicillin+Gentamicin, Amoxicillin+Cefotaxime, or Augmentin+Gentamicin).

### 2.4 Covariables

The following covariables were assessed as potential confounders for the relation between early‐life antibiotics and growth until age five years; sex, birth mode, maternal hypertension and infection during pregnancy, duration of ruptured membranes, percentile birth weight, maternal age, siblings < 5 years, breastfeeding ≥ 3 months and additional parent or GP reported antibiotic use ≤ 12 months and over the first five years of life (Table S1; set 1). To assess factors contributing to growth at age five years, we included set 1 variables and additional dietary and physical activity habits, which were collected at age five years only (Table S1; set 2). Data on dietary habits included fruit (≥ 1.5 pieces per day), vegetables (≥ 2 serving spoons per day), bread (slices per week) and lemonade (glasses per week) intake. Lemonade was defined as high sugar containing drinks made from diluted syrup or juice. Data on carbonated soda were available, but not included in the analyses, as consumption was rare (only 5% of parents reported weekly consumption, often less than one glass). Physical activity includes variables regarding screen time (minutes per day) and physical activity moments (either outdoors, at the sport club or at school).

### 2.5 Data Analysis

All analyses were performed in R version 4.4.1; *p*‐values < 0.05 were considered significant.

Differences in cohort characteristics between antibiotic‐exposed and non‐exposed children were assessed using descriptive statistics (chi‐square tests, Fisher's exact test, *t*‐test, or Kruskal‐Wallis test where appropriate).

We performed linear mixed models for weight‐for‐age, height‐for‐age, and weight‐for‐height *z*‐scores, including a random intercept per participant for repeated measures and a fixed effect for time as a factor. Covariables were included when they changed the estimates for early‐life antibiotic exposure by ≥ 10%. For antibiotic exposure after the first week of life, the final models were corrected for parent‐ and not GP‐reported antibiotics where relevant to reduce the number of missing data in the model. However, to ensure the robustness of the findings, we also replaced parent‐reported antibiotics with GP‐reported antibiotics in the final model in a post hoc fashion. Results were only reported if different from the parent‐reported model. When only GP‐reported antibiotic use met the ‘≥ 10% change in the estimate for early‐life antibiotic use’ condition, we included GP‐reported antibiotics in the model. Effect modification was assessed by including an interaction term to investigate potential interactions between age and week 1 antibiotic exposure. We then performed stratified analysis for the interval from birth until age ≤ 1 year and age > 1 year. Next, we replaced receiving any early‐life antibiotics with the antibiotic regimen in the linear mixed models to assess the relationship between different antibiotic regimens and growth outcomes over the first five years. Additionally, we performed post hoc analyses to compare the effect between different antibiotic regimens on growth outcomes, using pairwise comparisons in the emmeans‐package [25] and Benjamini‐Hochberg corrections for multiple testing.

To rule out confounding by underlying infection, we performed a sensitivity analysis, including only infants receiving antibiotics for ≤ 72 h. This group reflects cases where the medical team considered early‐onset neonatal sepsis unlikely, based on clinical signs, laboratory, and culture results. We performed a separate sensitivity analysis, excluding infants whose mothers received antepartum antibiotics, as this could confound neonatal regimen‐specific analyses and is collinear with early‐life antibiotic exposure.

Last, we developed an association model using linear regression and a backwards selection method. All covariables (Table S1 sets 1 and 2) with a *p*‐value < 0.2 in univariable analyses were included. For all analyses, in case of missing data, we used a complete case analysis.

### 2.6 Ethics and Data Availability

Informed consent was obtained from all caregivers, and both the ZEBRA and MUIS studies were approved by the Dutch National Ethics Committee (International Clinical Trial Registry Platform NL4882 and NL3821). The code used for data analysis is available at gitlab.com/fvanbeveren/mz_growth.

## 3 Results

### 3.1 Study Population

**FIGURE 1:**
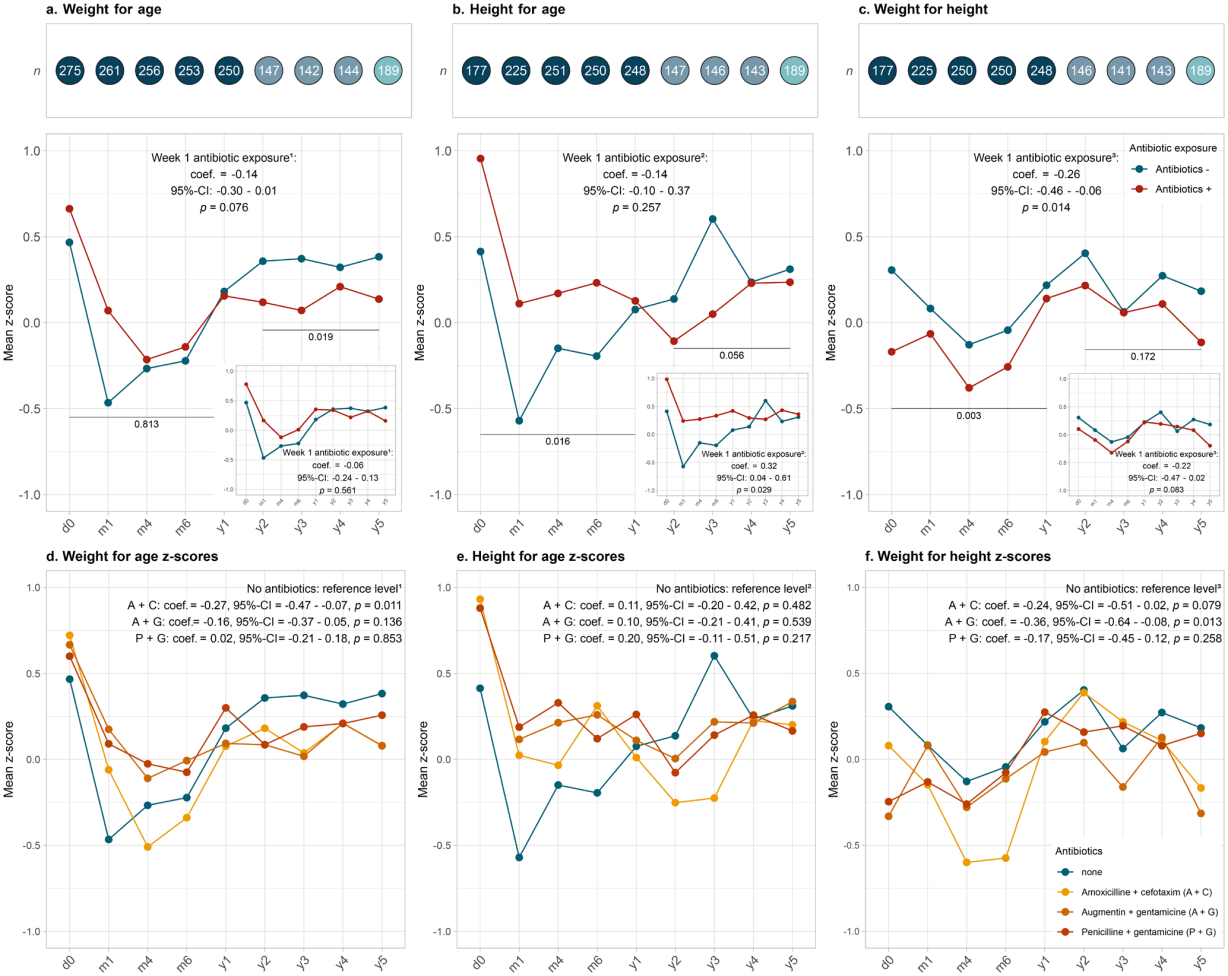
The effect of antibiotics in the first week of life on weight‐for‐age, height‐for‐age, weight‐for‐height. Growth (weight‐for‐age, height‐for‐age, weight‐for‐height) was explored from birth until the age of 5 years for children exposed to antibiotics (red) in the first week of life and non‐exposed children (blue). The x‐axis represents age in days (d), months (m) and years (y) and the y‐axis represents the mean *z*‐score (a–e). (a–c) The top of the figure shows the total number of study participants of which information about weight and height is available at different time points (these numbers also represent the total number of study participants with available information in figure d–f). Results from (stratified) linear mixed models are printed in the plots for weight‐for‐age, height‐for‐age and weight‐for‐height respectively. The results of sensitivity analysis excluding children receiving antibiotics > 72 h are plotted in the bottom right corner. (d–f) Shows the mean *z*‐scores over time per antibiotic regimen group (None; blue, Amoxicillin + Cefotaxime (A + C); yellow, Augmentin + Gentamicin (A + G); orange or Penicillin + Gentamicin (P + G); red) for weight‐for‐age, height‐for‐age and weight‐for‐height respectively. Results from linear mixed models are printed in the plots. ^1^Corrected for percentile birth weight, total weeks of gestational age, presence of siblings < 5 years, maternal age, duration of ruptured membranes, parent‐reported antibiotics ≤ 12 months; ^2^corrected for total weeks of gestational age, presence of siblings < 5 years, maternal age, parent‐reported antibiotics ≤ 12 months; ^3^corrected for total weeks of gestational age, duration of ruptured membranes, general practitioner reported antibiotics ≤ 12 months. Ns = non‐significant; coef. = coefficient; CI = confidence interval; *p* = *p*‐value.

In total 275 children were included, 147 with systemic antibiotics in the first week of life (ZEBRA) and 128 controls (MUIS) (Figure S1). At birth, growth data were available for 275 (weight‐for‐age) and 177 (height‐for‐age and weight‐for‐height) children, and for 250 and 189 children at one and five years respectively (Figure 1). Baseline characteristics are described in Table 1 and Table S2. Of the children treated for early‐onset neonatal sepsis, only two ultimately had positive blood cultures. Briefly, the cohorts differed in relation to delivery mode (vaginal birth 60.2% and 78.9% respectively, *p* = 0.001), maternal age (mean age 33.9 and 31.9 respectively, *p* < 0.001), having siblings < 5 years (56.9 and 23.8% respectively, *p* < 0.001), duration of ruptured membranes (median hours 3.0 and 11.0 respectively, *p* < 0.001), and antepartum maternal antibiotic treatment (2.4 and 44.2% respectively, *p* < 0.001). Antepartum maternal antibiotic regimens often included Amoxicillin and Gentamycin or a Cephalosporin. As several of these factors may act as confounders, we included these parameters in our covariable set (Table S1; set 1). To test for potential confounding effects of maternal antepartum antibiotic exposure, we performed sensitivity analyses to validate the models below, as this variable was collinear with early‐life antibiotic exposure.

**TABLE 1:** Cohort characteristics.

*n*	Antibiotics−	Antibiotics+	*p*
	128	147	
	*n* (%)^a^/Median [IQR]	*n* (%)^a^/Median [IQR]	
General			
Gender: Female	67 (52.3)	69 (46.9)	0.439
Siblings < 5 years present: yes	70 (56.9)	34 (23.8)	< 0.001
Parental factors			
Parental educational level			0.931^b^
Primary and secondary education	1 (0.8)	2 (1.4)	
Secondary vocational education	28 (23.0)	31 (21.8)	
University of Applied Sciences	43 (35.2)	46 (32.4)	
University	50 (41.0)	63 (44.4)	
Country of birth: The Netherlands			
Mother	106 (86.2)	119 (82.1)	0.455
Father	107 (87.0)	126 (89.4)	0.685
Maternal age (mean (SD))	33.93 (4.18)	31.86 (4.28)	< 0.001
Pre‐ and perinatal factors of newborn			
Infection during pregnancy: yes	21 (17.1)	22 (15.2)	0.798
Antepartum antibiotic exposure: yes	3 (2.4)	65 (44.2)	< 0.001
Duration ruptured membranes in hours (median[IQR])	3.00 [0.00, 6.75]	11.00 [5.00, 20.00]	< 0.001
Birth mode: Vaginal	77 (60.2)	116 (78.9)	0.001
Gestational age in weeks (mean (SD))	39.52 (1.12)	40.11 (1.45)	< 0.001^**c**^
Birth weight percentile (median [IQR])	60.00 [33.50, 78.00]	58.00 [35.00, 80.00]	0.877^d^
Birth weight categories			0.584
Dysmature (< p10)	13 (10.2)	12 (8.2)	
Normal (Low, p10‐p50)	38 (29.9)	51 (34.7)	
Normal (High, p50‐p90)	58 (45.7)	58 (39.5)	
Macrosome (> p90)	18 (14.2)	26 (17.7)	
Week 1 antibiotic use in days (median [IQR])		2.99 [2.37, 5.47]	
Antibiotic regimen for suspected early‐onset neonatal sepsis			< 0.001
None	128 (100.0)	0 (0.0)	
Amoxicilline + Cefotaxime	0 (0.0)	49 (33.3)	
Amoxicillin/Clavulanic Acid + Gentamicin	0 (0.0)	49 (33.3)	
Penicillin + Gentamicin	0 (0.0)	49 (33.3)	
Postnatal exposures			
Duration breastfeeding in days			0.610
0–7 days	34 (27.9)	33 (23.6)	
7–90 days	31 (25.4)	45 (32.1)	
90–180 days	18 (14.8)	17 (12.1)	
≥ 180 days	39 (32.0)	45 (32.1)	
Parent reported systemic antibiotics (Age > 7 days – 12 months): yes	36 (28.1)	23 (15.6)	0.018
Sum parent reported systemic antibiotics courses ≤ 5 years			0.110
0	81 (63.3)	104 (70.7)	
1	24 (18.8)	31 (21.1)	
2	12 (9.4)	7 (4.8)	
≥ 3	11 (8.6)	5 (3.4)	

*Note:* Describes the characteristics of the two combined birth cohorts. Antibiotics− = MUIS: 128 children that did not receive antibiotics directly postpartum; Antibiotics+ = ZEBRA: 147 children who received antibiotics directly postpartum.

Abbreviations: IQR, interquartile range; *n*, number; SD, standard deviation.

^a^ Numbers represent *n* (%) or the median and [interquartile range (IQR)].

^b^ Fisher's exact test.

^c^ *t*‐test.

^d^ Kruskal‐wallis test.

### 3.2 The Association Between Early‐Life Antibiotics and Growth Parameters Over the First Five Years of Life

No significant association was found between early‐life antibiotics and weight‐for‐age and height‐for‐age *z*‐scores across the entire first five years of life (Figure 1a,b). Given the change in direction of the effect of early‐life systemic antibiotics, we included an interaction term with time and found a significant difference in the effect of early‐life antibiotics on weight‐for‐age, height‐for‐age, and weight‐for‐height *z*‐scores for different timepoints (Table S3). Stratified analyses for the period until age 1 year showed no significant difference in weight‐for‐age *z*‐scores between antibiotic‐exposed and non‐exposed children, but height‐for‐age *z*‐scores were on average 0.32 higher for antibiotic‐exposed children (95%‐CI = 0.06 to 0.57; *p* = 0.016; Table S4). Conversely, stratified analyses for children aged > 1 year showed that children who received early‐life systemic antibiotics had on average 0.33 lower weight‐for‐age (95%‐CI = −0.60 to −0.06; *p* = 0.019) and similar height‐for‐age *z*‐scores (95%‐CI = −0.65 to 0.00; *p* = 0.056; Table S4). Weight‐for‐height *z*‐scores were lower across the first five years in children who received early‐life systemic antibiotics (Figure 1c), with on average a 0.26 lower weight‐for‐height *z*‐score compared to unexposed children (95%‐CI: −0.46 to −0.06; *p* = 0.014). Stratified analysis showed on average 0.31 lower weight‐for‐height *z*‐scores for the interval from birth until age ≤ 1 year (95%‐CI = −0.51 to −0.11; *p* = 0.003) and on average 0.17 lower *z*‐scores for age > 1 year (95%‐CI = −0.41 to 0.07; *p* = 0.172; Table S4). Sensitivity analyses excluding children receiving antibiotics > 72 h showed consistent results to the analyses above (Figure 1a–c), and excluding all children exposed to antepartum antibiotics also did not change our findings (data not shown).

### 3.3 Influence of Different Antibiotic Regimens on Growth Parameters

Longitudinal analysis of different antibiotic regimens on growth patterns over the first five years revealed that children treated with Amoxicillin+Cefotaxime had on average 0.27 lower weight‐for‐age *z*‐scores compared to untreated children (Figure 1d). Comparisons between the distinct antibiotic regimen groups showed no difference in weight‐for‐age *z*‐scores. No significant differences were found for height‐for‐age *z*‐scores (Figure 1e).

The largest effect was seen in weight‐for‐height scores, with children treated with Amoxicillin+Cefotaxime showing on average 0.24 lower *z*‐scores. Similarly, children treated with Augmentin+Gentamicin had on average 0.36 lower weight‐for‐height *z*‐scores, and those treated with Penicillin+Gentamicin had on average 0.17 lower *z*‐scores compared to untreated children (Figure 1f). Post hoc comparison for the differential effects of the regimens on weight‐for‐height *z*‐scores showed again no direct differences between the regimens. Sensitivity analysis, excluding all children exposed to antepartum antibiotics, showed that despite a reduced power of the model, (1) the association between Augmentin+Gentamicin and reduced weight‐for‐height *z*‐scores was maintained, (2) the association of Amoxicillin+Cefotaxime with both weight‐for‐age and weight‐for‐height *z*‐scores was no longer significant, and (3) Penicillin+Gentamicin exposure was significantly associated with lower weight‐for‐height *z*‐scores (Table S5).

### 3.4 Factors Influencing Weight and Height at Age Five Years

**FIGURE 2:**
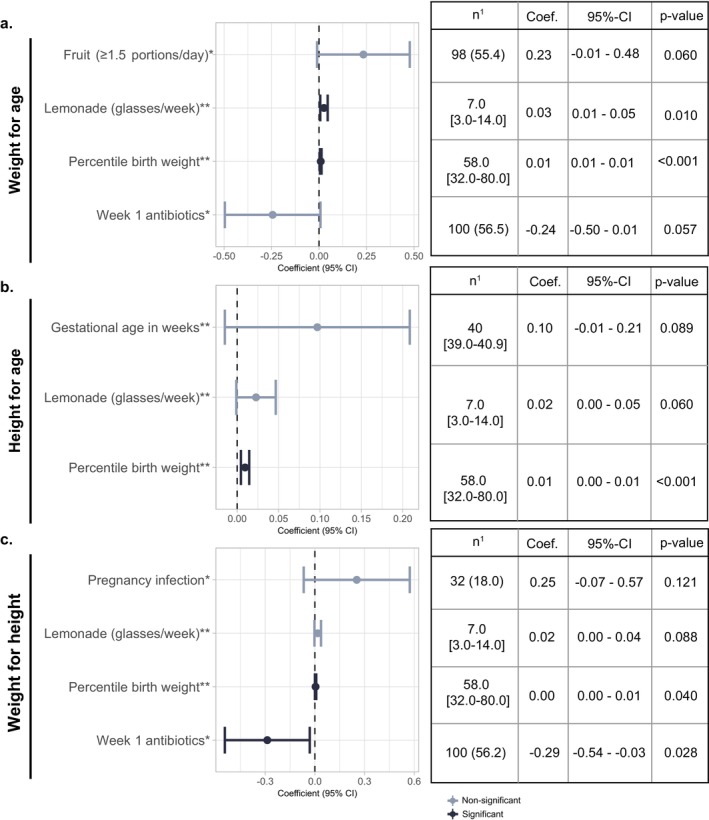
Factors contributing to weight‐for‐age, height‐for‐age, and weight‐for‐height *z*‐scores at age 5 years. (a–c) A backwards selected linear regression model was used to study covariables that potentially affect growth parameters at age 5 years for weight‐for‐age (a), height‐for‐age (b) and weight‐for‐height (c). Covariables with univariable *p*‐value < 0.2 were included in the full model. Final models are presented per growth outcome. The y‐axis represents the coefficient with its corresponding 95% Confidence Interval (CI). A significant difference is depicted in black and non‐significant results in grey. More detail can be found in the corresponding tables to the right of the image. *Response is yes; **Continuous variable. ^1^numbers represent *n* (%) or the median and [inter quartile range (IQR)].

At age five years, we assessed additional factors contributing to weight‐for‐age, height‐for‐age, and weight‐for‐height *z*‐scores using linear regression models and a backwards selection method (Table S1; sets 1 and 2). Besides exposure to systemic antibiotics in the first week of life showing 0.24 lower weight‐for‐age *z*‐scores at age 5 years (95%‐CI: −0.50 to 0.01; *p* = 0.058; Figure 2a), corresponding to approximately 550 g lower weight, the percentile birth weight, the weekly number of glasses of lemonade, and daily ≥ 1.5 pieces of fruit were associated with higher weight‐for‐age *z*‐scores at age 5 years (Figure 2a). At age five years, percentile birth weight was also significantly associated with a higher height‐for‐age *z*‐score (95%‐CI 0.00 to 0.01; *p* < 0.001 Figure 2b), whereas the total weeks of gestational age and the weekly number of glasses of lemonade showed a trend towards higher height‐for‐age *z*‐scores (Figure 2b). A 0.29 lower weight‐for‐height *z*‐scores at age five years was found in children who received antibiotics in their first week of life (95%‐CI: −0.54 to −0.03; *p* = 0.028; Figure 2c). Conversely, the percentile birth weight was associated with higher weight‐for‐height *z*‐scores (Figure 2c).

## 4 Discussion

In this study, we investigated the association between antibiotic exposure in the first days of life and growth patterns until age five years. We found that children exposed to early‐life antibiotics had significantly higher height‐for‐age *z*‐scores until age one year and lower weight‐for‐age *z*‐scores between one and five years, resulting in lower weight‐for‐height *z*‐scores on average over the first five years of life. As weight‐for‐height can be regarded as a composite outcome of weight‐for‐age and height‐for‐age, our results indicate that the effect of early‐life antibiotics on lower weight‐for‐height *z*‐scores is primarily driven by higher height‐for‐age *z*‐scores in the first year and lower weight‐for‐age *z*‐scores after the first year of life. A more detailed analysis of different antibiotic regimens suggests that especially a combination of Augmentin + Gentamicin is related to lower weight‐for‐height *z*‐scores across the first five years of life, and that this effect is independent of antepartum antibiotic exposure. A cross‐sectional analysis at age five years assessed the combined impact of epidemiological, peri‐, postnatal, diet, and physical activity exposures on growth parameters, which showed a persistent effect of early‐life antibiotics on lower weight‐for‐height *z*‐scores. Additionally, a higher percentile birth weight remained related to higher levels of all three growth parameters, whereas increased weekly lemonade consumption at age five years was linked to higher weight‐for‐age *z*‐scores at this age.

The finding that antibiotic exposure in the first week of life is associated with reduced growth over the first five years of life is confirmed by other studies showing reduced growth at age one [22] and at age six years in boys [16]. In our study, gender was no confounding factor. Interestingly, antibiotic exposure past the neonatal period has previously been related to overweight and obesity later in childhood. This effect has been shown in several clinical studies and is most evident when children were exposed to repeated treatments, broad‐spectrum antibiotics, and treatment within the first six months of life [17, 18, 19, 20, 21, 26]. Other studies also show an effect of prenatal or intrapartum antibiotics on weight gain during childhood, but these associations were small [20, 27, 28, 29, 30]. In our dataset, the number of mothers with antepartum antibiotic exposure was too small to study this as a separate variable. Moreover, this covariable was colinear with early‐life antibiotic exposure. Therefore, we performed a sensitivity analysis instead, showing that neonatal antibiotic exposure was associated with reduced growth irrespective of maternal intrapartum antibiotic use. Taken together, the association between antibiotic exposure and growth outcomes might be dependent on the timing of antibiotic exposure and the number of antibiotic courses.

An additional factor that seems to be important for the relation between antibiotics and growth is the type of regimen. One previous clinical study, comparing the effects of Amoxicillin+Gentamicin, Augmentin+Gentamicin, and Penicillin+Gentamicin administration during the first week of life on growth until age one year, found that Amoxicillin+Gentamicin had the largest effect on both weight and height [22]. We find lower weight‐for‐height *z*‐scores in Augmentin+Gentamicin exposed children, independent of antepartum antibiotic exposure, across the first five years of life. The effects of the other two neonatal antibiotic regimen groups (i.e., Amoxicillin+Cefotaxime and Penicillin+Gentamicin) shifted when excluding children exposed to antepartum antibiotics. There are several potential explanations for these shifts; first, the effect of the individual neonatal antibiotic regimens might be masked via exposure to (another type of) antepartum antibiotic regimen. Second, the shift may be explained by an opposite effect on growth of antepartum and neonatal antibiotic exposure (i.e., increase instead of decrease), as discussed above [20, 27, 28, 30]. This could explain why we observed lower weight‐for‐height *z*‐scores in the Penicillin+Gentamicin group when excluding antepartum antibiotic‐exposed children, but not in the entire cohort (e.g., the effect was masked), and why we observed a loss of effect in the Amoxicillin+Cefotaxime group after excluding the antepartum antibiotic exposed children (e.g., effect attributed (in part) to antepartum rather than early life antibiotics).

One physiological mechanism that could link early‐life antibiotics to altered growth patterns, is antibiotic‐induced alterations of the gut microbiome. In this cohort antibiotic‐exposed children previously showed a decreased abundance of *Bifidobacterium* and an increase in *Klebsiella* and *Enterococcus* species compared to controls [15]. In the current study, the most consistent effects on growth were observed in Augmentin+Gentamicin exposed children, for which we also found the most pronounced reduction in microbial diversity in the first year of life [15]. Other studies have found an association the gut microbiome and growth patterns. For example, reduced microbial diversity and lower abundance of *Faecalibacterium, Ruminococcus* and *Roseburia* at one year was associated with lower weight gain between ages one to five [31] and lower levels of *Bifidobacterium spp* after early‐life antibiotics with reduced growth until age six years [16]. Alternatively, lower *Bifidobacterium* spp. levels at age 2.5 years have been associated with overweight and obesity at this age after antibiotic exposure in the first year of life [32]. Antibiotic‐induced alterations in the gut microbiome composition have been shown to affect the host‐metabolism. In mice, antibiotic‐induced microbiome disruptions lead to a reduction in butyrate levels, a short‐chain fatty acid, making enterocytes use glucose instead of butyrate as energy source [33]. The shift in the use of glucose instead of butyrate as energy source may affect the glucose homeostasis throughout the body and consequently impact growth patterns. This is further supported by the finding that antibiotics reduced weight in specific‐pathogen free mice, but not in germ‐free mice [34] and that faecal microbiota transplants from antibiotic‐exposed infants in the neonatal period to germ‐free mice resulted in impaired growth, though only in male mice [16]. Conversely, in mice treated with subtherapeutic doses of oral antibiotics an increased fat mass and short‐chain fatty acid were found [35]. Taken together with the epidemiological data, this suggests that the antibiotic dosage and timing of antibiotic‐induced microbiome disruption may affect child growth in an opposing manner.

Our study has certain limitations. First, two different birth cohorts were used that enrolled children in different years and had different initial objectives. Although the cohorts were set up in the same geographic region by the same study team and using similar study protocols and enrollment criteria (except for the suspected early‐onset neonatal sepsis criterium), minor confounding factors related to environmental exposures may still affect the outcomes. Even so, we assessed and corrected for differences in baseline characteristics when these impacted the relation between early life antibiotics and growth outcomes. Therefore, potential differences arising from slight variability in the study designs can be considered negligible. Second, due to the study design, participant numbers dropped after the first year of life, which may have led to statistical bias. Third, parental weight and height data were unavailable, which could have helped in estimating and correcting for the genetic drivers of a child's growth trajectory. Lastly, these studies were not powered to study growth as a primary aim: the primary aim of the ZEBRA cohort was to study the effects of antibiotics on microbiome development and that of the MUIS cohort to study the development of the microbiome in healthy newborns in relation to mode of delivery. Despite this, we believe that the current analysis provides us with valuable information on growth patterns in relation to antibiotic exposure to different regimens. Strengths of this study include the extensive data on potential confounders and the ability to investigate the impact of different antibiotic regimens on the child's growth over the first five years of life.

Overall, the data from our study support the idea that early‐life antibiotic exposure is associated with childhood growth. Our findings indicate that antibiotics administered within the first week of life are associated with reduced weight‐for‐height until age five years, with the regimen of Augmentin + Gentamicin showing the most consistent effects on growth. Since antibiotic treatment in early life can be essential in certain cases, clinicians should consider the choice of regimen carefully, and diagnostic tools for appropriate initiation of antibiotics for early‐onset neonatal sepsis are required to prevent overtreatment. Furthermore, understanding the complex relationship between early childhood antibiotic use and its effects on growth is essential for developing guidelines that minimise adverse outcomes while effectively managing bacterial infections.

## Author Contributions

G.J.v.B. and M.A.G.P. pre‐processed and analyzed the data. L.M.v.L. and G.J.v.B. visualized the data. L.M.v.L., G.J.v.B., M.A.G.P., D.S., and S.E. assisted in the statistical analyses. L.M.v.L., G.J.v.B., D.S., S.E., D.B., and M.A.v.H. aided in the interpretation of the data. L.M.v.L. and G.J.v.B. wrote the original draft of the manuscript. D.B. and M.A.v.H. were responsible for conceptualizing the study, acquiring funding, and supervision. M.A.v.H. supervised the data acquisition. All authors critically revised and approved the manuscript before submission.

## Consent

All caregivers of participants provided informed consent to participation.

## Conflicts of Interest

The authors declare no conflicts of interest.

## Supporting information


**Data S1:** apa70322‐sup‐0001‐supinfo.docx.

## Data Availability

The data that support the findings of this study are available on request from the corresponding author. The data are not publicly available due to privacy or ethical restrictions.
