# Supplementary material for: Aberrant Growth in 5‐Year‐Old Children After Antibiotics in the First Week of Life
Source: Acta Paediatr. 2025 Oct 7;115(2):309–18. doi: 10.1111/apa.70322 (PMC12794745; doi:10.1111/apa.70322)
Supplement: Supplementary file 1 — Data S1: apa70322‐sup‐0001‐supinfo.docx. [file APA-115-309-s001.docx]

**Supplementary data to “Aberrant growth in 5-year-old children after antibiotics in the first week of life”**

Authors:

Lisanne M. van Leeuwen^1, §^

Gina J. van Beveren^1,2, §^

Marieke A. G. Peeters^1^

Dennis Souverein^3^

Sjoerd Euser^3^

Debby Bogaert^2, 4, #, *^

Marlies A. van Houten^1, #^

^§^These authors contributed equally and share first authorship

^#^These authors jointly supervised the work, contributed equally and share senior authorship

Institutions:

^1^Department of Paediatrics, Spaarne Hospital, Haarlem, The Netherlands.

^2^Department of Paediatric Immunology and Infectious Diseases, Wilhelmina Children’s Hospital/University Medical Center Utrecht, Utrecht, The Netherlands.

^3^Regional Public Health Laboratory Kennemerland, Haarlem, The Netherlands

^4^Centre for Inflammation Research, Institute for Regeneration and Repair, University of Edinburgh, Edinburgh, United Kingdom

**Supplementary figures**

Figure S1 | numbers per timepoint for different antibiotic groups

**Supplementary tables**

Table S1 | Covariable sets

Table S2 | Additional baseline information

Table S3 | Linear mixed model results with interaction terms

Table S4 | Stratified linear mixed model analyses

Table S5 | Sensitivity analysis antibiotic regimens

**
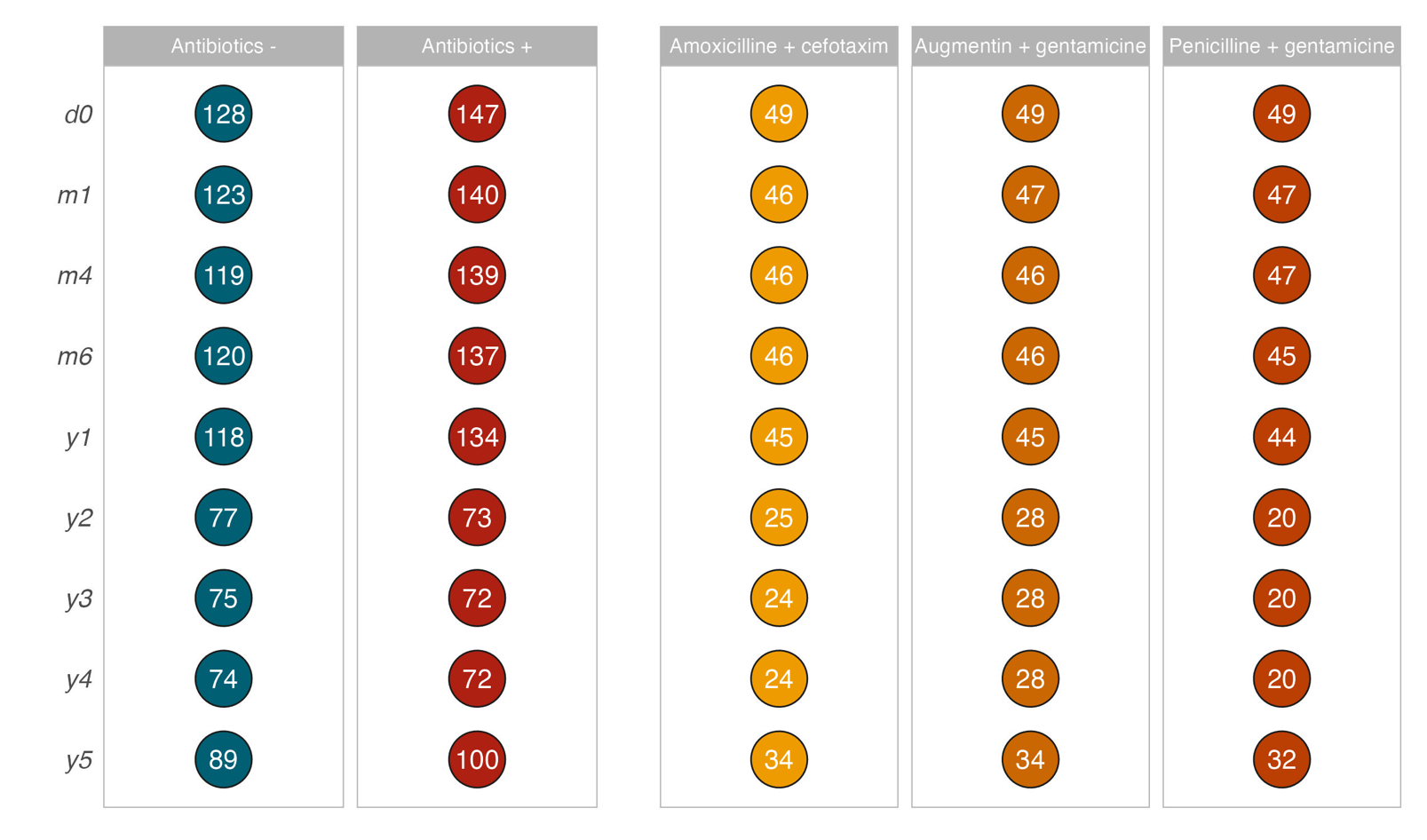
Supplementary figures**

Figure S1 | numbers per timepoint for different antibiotic groups

Number of children in the study at each timepoint, stratified for children exposed to antibiotics in the first week of life (Antibiotics +) and non-exposed children (Antibiotics -). The numbers for children exposed to antibiotics were further stratified for the different antibiotic regimens. d = day; m = month; y = year.

**Supplementary tables**

| **Table S1: covariables** | | |
| --- | --- | --- |
| Group | Set 1 | Set 2 |
| General | Sex;  Percentile birth weight;  Total weeks of gestational age;  Siblings <5 years;  Maternal age |  |
| Pre/perinatal exposure | Birth mode;  Pregnancy induced hypertension yes/no  Duration of ruptured membranes (hours)  Pregnancy infection |  |
| Postnatal exposure | Breastfeeding ≥3 months yes/no  Antibiotic courses ≤12m yes/no^1^  Antibiotic courses ≤5y (0, 1, 2 or ≥3)^1^ |  |
| Diet |  | Fruit (≥ 1.5 pieces/day);  Vegetables (≥2 serving spoons/day);  Bread (slices/week);  Lemonade (glasses/week) |
| Physical activity |  | Screen time (min/day);  Sleep (hours/night);  Falling asleep within 20 min;  Activity (moments/week)^2^ |

Table S1 | Covariables

^1^Either parent- or general practitioner reported antibiotic use. Parent-reported antibiotic use was used instead of general practitioner data, to reduce loss of missing data. When only general practitioner reported antibiotic use changed the estimate of week 1 antibiotic use with ≥10%, we corrected for general practitioner reported antibiotic use. ^2^An activity moment is defined as exercise at school, sport association or playing outdoors. m=months; y= years; min=minute

| **Table S2: Additional cohort characteristics** | | | |
| --- | --- | --- | --- |
|  | **Antibiotics -** | **Antibiotics +** | **p** |
| n | 128  N (%)^a^ / Median [IQR] | 147  N (%)^a^ / Median [IQR] |  |
| Epidemiological / environmental factors | | | |
| Smoking in house ≤12m: yes | 3 (2.3) | 1 (0.7) | 0.348^b^ |
| Household pets | 56 (45.9) | 60 (40.8) | 0.475 |
| Maternal factors during pregnancy and delivery | | | |
| Pregnancy induced hypertension | 7 (5.5) | 8 (5.4) | 1.000 |
| Perinatal factors of newborn | | | |
| Season of birth |  |  | 0.143 |
| Winter | 27 (21.1) | 27 (18.4) |  |
| Spring | 28 (21.9) | 32 (21.8) |  |
| Summer | 47 (36.7) | 41 (27.9) |  |
| Autumn | 26 (20.3) | 47 (32.0) |  |
| Dietary characteristics at age 5 years | | | |
| Glasses of lemonade per week | 8.75 [4.00, 14.00] | 5.00 [1.75, 10.50] | <0.001^c^ |
| ≥ 1.5 pieces of fruit per day: yes | 40 (50.0) | 59 (59.0) | 0.291 |
| ≥ 2 serving spoons of vegetables | 24 (29.6) | 26 (26.3) | 0.738 |
| Whole wheat bread intake |  |  | 0.839 |
| Max 1 /week | 5 (6.2) | 9 (9.1) |  |
| 2-3 /week | 5 (6.2) | 8 (8.1) |  |
| 4-5 /week | 10 (12.3) | 12 (12.1) |  |
| 6-7 /week | 61 (75.3) | 70 (70.7) |  |
| Level of activity at age 5 years | | | |
| Screen time / day |  |  | 0.348 |
| < 45 min. | 24 (29.6) | 25 (25.0) |  |
| 45 – 60 min. | 18 (22.2) | 27 (27.0) |  |
| 60 – 90 min. | 23 (28.4) | 36 (36.0) |  |
| > 90 min. | 16 (19.8) | 12 (12.0) |  |
| Exercise moments / week | 9.00 [6.75, 10.00] | 8.00 [6.00, 10.00] | 0.960 |
| Sleep duration per night in hours | 11.5 [11.5, 11.75] | 11.5 [11.25, 12.00] | 0.698 |
| Falling asleep within 20 min | 70 (86.4) | 89 (89.0) | 0.765 |
| Medication use | | | |
| GP reported systemic antibiotics (Age >7 days – 12 months) | 18 (18.9) | 15 (16.5) | 0.804 |
| Sum GP reported systemic antibiotics courses≤ 5 years |  |  | 0.648 |
| 0 | 44 (46.3) | 45 (49.5) |  |
| 1 | 26 (27.4) | 29 (31.9) |  |
| 2 | 14 (14.7) | 9 (9.9) |  |
| ≥3 | 11 (11.6) | 8 (8.8) |  |
| Parent reported inhalation corticosteroid use | 3 (2.3) | 4 (2.7) | 1.00^b^ |
| GP reported inhalation corticosteroid use |  |  | 0.599^b^ |
| 0 times | 90 (94.7) | 88 (96.7) |  |
| 1 times | 4 (4.2) | 1 (1.1) |  |
| 2 times | 1 (1.1) | 1 (1.1) |  |
| 3 times | 0 (0.0) | 1 (1.1) |  |
| GP reported topical corticosteroid use |  |  | 0.363 |
| 0 times | 74 (77.9) | 59 (64.8) |  |
| 1 times | 12 (12.6) | 17 (18.7) |  |
| 2 times | 5 (5.3) | 6 (6.6) |  |
| 3 times | 3 (3.2) | 4 (4.4) |  |
| 4 times | 1 (1.1) | 1 (1.1) |  |
| 5 times | 0 (0.0) | 2 (2.2) |  |
| 7 times | 0 (0.0) | 2 (2.2) |  |

Table S2 | Additional cohort characteristics

^a^numbers represent n (%) or the median and [inter quartile range (IQR)]; ^b^Fisher’s exact test; ^b^cKruskal-wallis test. n = number, m = months; min = minutes; GP = general practitioner.

| **Table S3: linear mixed models with interaction term** | | | | | | | | | |
| --- | --- | --- | --- | --- | --- | --- | --- | --- | --- |
|  | Weight-for-age^a^ | | | Height-for-age^b^ | | | Weight-for-height^c^ | | |
| term | coef. | 95%-CI | p | coef. | 95%-CI | p | coef. | 95%-CI | p |
| Week 1 antibiotics = yes | 0.00 | -0.21 - 0.21 | 0.975 | 0.51 | 0.17 - 0.85 | 0.003 | -0.76 | -1.12 - -0.41 | <0.001 |
| Week 1 antibiotics* Age: <7 days | Ref. | Ref. | Ref. | Ref. | Ref. | Ref. | Ref. | Ref. | Ref. |
| Week 1 antibiotics * Age: m1 | 0.35 | 0.13 - 0.57 | 0.002 | 0.14 | -0.20 - 0.48 | 0.428 | 0.64 | 0.24 - 1.04 | 0.002 |
| Week 1 antibiotics* Age: m4 | -0.20 | -0.42 - 0.02 | 0.078 | -0.25 | -0.58 - 0.08 | 0.137 | 0.35 | -0.04 - 0.74 | 0.084 |
| Week 1 antibiotics* Age: m6 | -0.13 | -0.35 - 0.09 | 0.250 | -0.15 | -0.48 - 0.18 | 0.385 | 0.42 | 0.03 - 0.81 | 0.037 |
| Week 1 antibiotics* Age: y1 | -0.23 | -0.45 - 0.00 | 0.049 | -0.51 | -0.84 - -0.18 | 0.003 | 0.64 | 0.25 - 1.03 | 0.001 |
| Week 1 antibiotics* Age: y2 | -0.35 | -0.61 - -0.08 | 0.010 | -0.81 | -1.19 - -0.43 | <0.001 | 0.71 | 0.29 - 1.14 | 0.001 |
| Week 1 antibiotics* Age: y3 | -0.44 | -0.71 - -0.17 | 0.001 | -1.13 | -1.51 - -0.75 | <0.001 | 0.84 | 0.41 - 1.26 | <0.001 |
| Week 1 antibiotics* Age: y4 | -0.30 | -0.56 - -0.03 | 0.030 | -0.62 | -1.00 - -0.24 | 0.001 | 0.59 | 0.17 - 1.02 | 0.006 |
| Week 1 antibiotics* Age: y5 | -0.51 | -0.76 - -0.27 | <0.001 | -0.68 | -1.03 - -0.33 | <0.001 | 0.42 | 0.03 - 0.82 | 0.036 |

Table S3 | Linear mixed models with interaction term

^a^corrected for percentile birth weight, total weeks of gestational age, presence of siblings <5 years, maternal age, duration of ruptured membranes, parent-reported antibiotics ≤12 months; ^b^corrected for total weeks of gestational age, presence of siblings <5 years, maternal age, parent-reported antibiotics ≤12 months; ^c^corrected for total weeks of gestational age, duration of ruptured membranes, general practitioner reported antibiotics ≤12 months.

A random intercept for participant identifier and an interaction term between week 1 antibiotic use and time (as factor) were included in all models. Additionally, models were corrected for covariables that changed the estimate for week 1 antibiotics with >10% (a-c). coef.; model coefficient; CI = confidence interval.

| **Table S4: Stratified linear mixed model analyses** | | | | | | | | | | | | | | | | | | |
| --- | --- | --- | --- | --- | --- | --- | --- | --- | --- | --- | --- | --- | --- | --- | --- | --- | --- | --- |
|  | Weight-for-age | | | | | | Height-for-age | | | | | | Weight-for-height | | | | | |
|  | Age ≤ 1 year^a^ | | | Age > 1 year^b^ | | | Age ≤ 1 year ^c^ | | | Age > 1 year ^b^ | | | Age ≤ 1 year ^d^ | | | Age > 1 year^e^ | | |
| term | coef. | 95%-CI | p | coef. | 95%-CI | p | coef. | 95%-CI | p | coef. | 95%-CI | p | coef. | 95%-CI | p | coef. | 95%-CI | p |
| Week 1 antibiotics = yes | -0.02 | -0.15 - 0.12 | 0.813 | -0.33 | -0.6 - -0.06 | 0.019 | 0.32 | 0.06 - 0.57 | 0.016 | -0.32 | -0.65 - 0.00 | 0.056 | -0.31 | -0.51 - -0.11 | 0.003 | -0.17 | -0.41 - 0.07 | 0.172 |

Table S4 | Stratified linear mixed model analyses

^a^corrected for percentile birth weight, total number of weeks of gestational age, presence of siblings <5 years, parent-reported antibiotic use ≤12 months. ^b^corrected for total number of weeks of gestational age, presence of siblings <5 years, duration of ruptured membranes. ^c^corrected for total number of weeks of gestational age, presence of siblings <5 years, duration of ruptured membranes, maternal age. ^d^corrected for total number of weeks of gestational age, duration of ruptured membranes, parent -reported antibiotic use ≤12 months. ^e^corrected for presence of siblings <5 years, duration of ruptured membranes, parent-reported sum of antibiotics over the first 5 years.

A random intercept for participant identifier was included and a fixed effect for time (as a factor) in all models. Additionally, models were corrected for covariables that changed the estimate for week 1 antibiotics with >10% (a-e). CI=confidence interval; coef. =model coefficient; p=p-value.

| **Table S5: Sensitivity analysis for antibiotic regimen** | | | | | | | | | |
| --- | --- | --- | --- | --- | --- | --- | --- | --- | --- |
|  | Weight-for-age^a^ | | | Height-for-age^b^ | | | Weight-for-height^c^ | | |
| term | coef. | 95%-CI | p | coef. | 95%-CI | p | coef. | 95%-CI | p |
| No antibiotics | Ref. | Ref. | Ref. | Ref. | Ref. | Ref. | Ref. | Ref. | Ref. |
| Amoxicilline + cefotaxim | -0.20 | -0.44- 0.04 | 0.112 | 0.05 | -0.32 - 0.42 | 0.798 | -0.23 | -0.53 - 0.07 | 0.149 |
| Augmentin + gentamicine | -0.21 | -0.48 - 0.06 | 0.138 | 0.03 | -0.37 - 0.43 | 0.883 | -0.52 | -0.88 - -0.16 | 0.007 |
| Penicilline + gentamicine | -0.09 | -0.37 - 0.18 | 0.516 | 0.40 | -0.03 - 0.82 | 0.074 | -0.62 | -1.02 - -0.23 | 0.003 |

Table S5 | Sensitivity analysis for antibiotic regimen

^a^corrected for percentile birth weight, total weeks of gestational age, presence of siblings <5 years, maternal age, duration of ruptured membranes, parent-reported antibiotics ≤12 months; ^b^corrected for total weeks of gestational age, presence of siblings <5 years, maternal age, parent-reported antibiotics ≤12 months; ^c^corrected for total weeks of gestational age, duration of ruptured membranes, general practitioner reported antibiotics ≤12 months.

A random intercept for participant identifier was included and a fixed effect for time (as a factor) in all models. Additionally, models were corrected for covariables that changed the estimate for week 1 antibiotics with >10% (a-c). coef. = model coefficient; CI = confidence interval.
